# Varicella Orchitis: A Case Report and Literature Review

**DOI:** 10.7759/cureus.66978

**Published:** 2024-08-16

**Authors:** Mohamed S Mohsin, Nader F Gaballa, Waleed Elsayed

**Affiliations:** 1 Urology, University Hospitals Birmingham NHS Foundation Trust, Birmingham, GBR

**Keywords:** mumps, testicular atrophy, chickenpox, varicella zoster virus, orchitis

## Abstract

Orchitis secondary to chickenpox (Varicella orchitis) is a rare sequelae of a common condition with only nine reports available from the current literature. We describe a case of orchitis secondary to chickenpox in a young male including the management and follow-up. In addition, the authors conducted a comprehensive review of the existing literature. In conclusion, orchitis secondary to chickenpox may lead to testicular atrophy, with potential implications for spermatogenesis, fertility and endocrine function yet to be established.

## Introduction

Orchitis refers to the inflammation of the testicle and is usually associated with inflammation of the epididymis (epididymo-orchitis) which presents as a gradual onset of swelling and pain [1]. This is in contrast to the acute onset and rapid progression of testicular pain seen in testicular torsion which results in loss of tissue if not surgically corrected. 

The most common cause of epididymo-orchitis in younger males (under the age of thirty five years) is a sexually transmitted infection secondary to Neisseria gonorrhoeae or Chlamydia trachomatis while the most common causative organism in older males is Escherichia coli [2]. On clinical examination, a tender, swollen epididymis which is more pronounced towards the tail of the epididymis initially in addition to a tender and swollen testicle. The management of bacterial epididymo-orchitis involves antibiotics targeted at the most likely causative organism depending on age, history and sexual activity in addition to symptomatic treatment with analgesia and a scrotal support. In instances where an abscess is suspected and confirmed with an ultrasound, intravenous antibiotics and surgical drainage of the abscess will be needed.

The rate of testicular atrophy following epididymo-orchitis in a study including 140 patients across all age groups was 1.4% with a rate of 5% for testicular loss and atrophy together [3]. In contrast to bacterial causes of epididymo-orchitis, the most common cause of viral orchitis is mumps while orchitis secondary to chickenpox is a rare sequelae or complication. We present a case report of a young male diagnosed with orchitis secondary to chickenpox as well as its management in order to broaden the existing body of knowledge surrounding chickenpox induced orchitis and its potential sequelae. The authors conducted a comprehensive literature review compare and understand the presentation, treatment and outcomes of chickenpox induced orchitis. 

## Case presentation

A 23-year-old male presented to the Department of Urology with a complaint of right hemiscrotal pain and swelling. He developed a generalised rash and was subsequently diagnosed with chickenpox 5 days before his attendance and at the time of his consultation, he had a polymorphic rash on his face and trunk in association with a fever. 

The right hemiscrotal pain gradually developed on the morning of his presentation and was associated with a mild swelling of the affected testicle. Apart from the discomfort, he remained well with no symptoms suggesting a urinary tract infection (UTI) or sexually transmitted disease (STD). 

**Figure 1:**
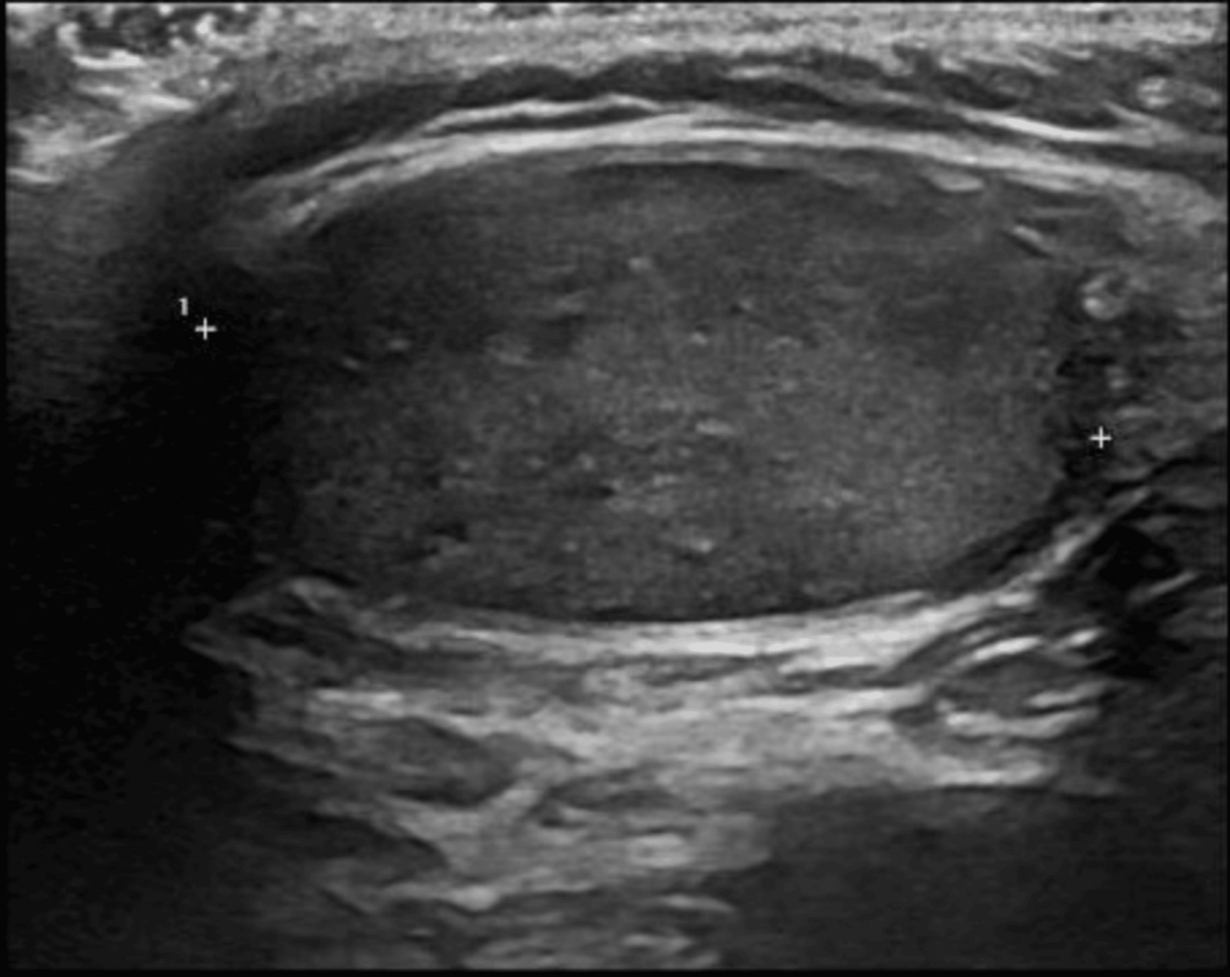
Right testicle (affected) measuring 39 mm in length with internal hypoechoic regions, suggesting infection.

On examination, he appeared systemically well with haemodynamic parameters within range. The right testicle was within the right hemi-scrotum and oriented vertically. However, it was swollen and moderately tender with no palpable masses. The right epididymis was slightly tender to palpation with no appreciable swelling. A cremasteric reflex was appreciated. The left (unaffected) hemi scrotum and its contents were unremarkable. Laboratory investigations showed a CRP of 65 (normal range: 0-5 mg/L) with a white blood cell count (WBC) of 13.2 (normal range: 3.26-11.20 x 10^9^/L.) In the context of active chickenpox, the patient was diagnosed with right-sided orchitis secondary to chickenpox. He was discharged from our unit with a two-week course of ciprofloxacin to provide adequate cover for a bacterial cause of orchitis and advised to wear a scrotal support for ten days as well as use paracetamol and ibuprofen for comfort. As testicular torsion was clinically ruled out, we arranged for an ultrasound scan of the testes one month following his presentation to assess for any intra-testicular pathology and the resolution of inflammation. Our patient continued to recover at home and did not re-attend our department between the first contact and his ultrasound scan. The ultrasound scan was done exactly one-month post-presentation and demonstrated a right testicle that was 39 mm x 19 mm x 37 mm in size with diffuse hypo-echoic changes within it (Figure 1). The testicular vasculature appeared normal with no intra-testicular lesions of note and a sonographically normal epididymis.

**Figure 2:**
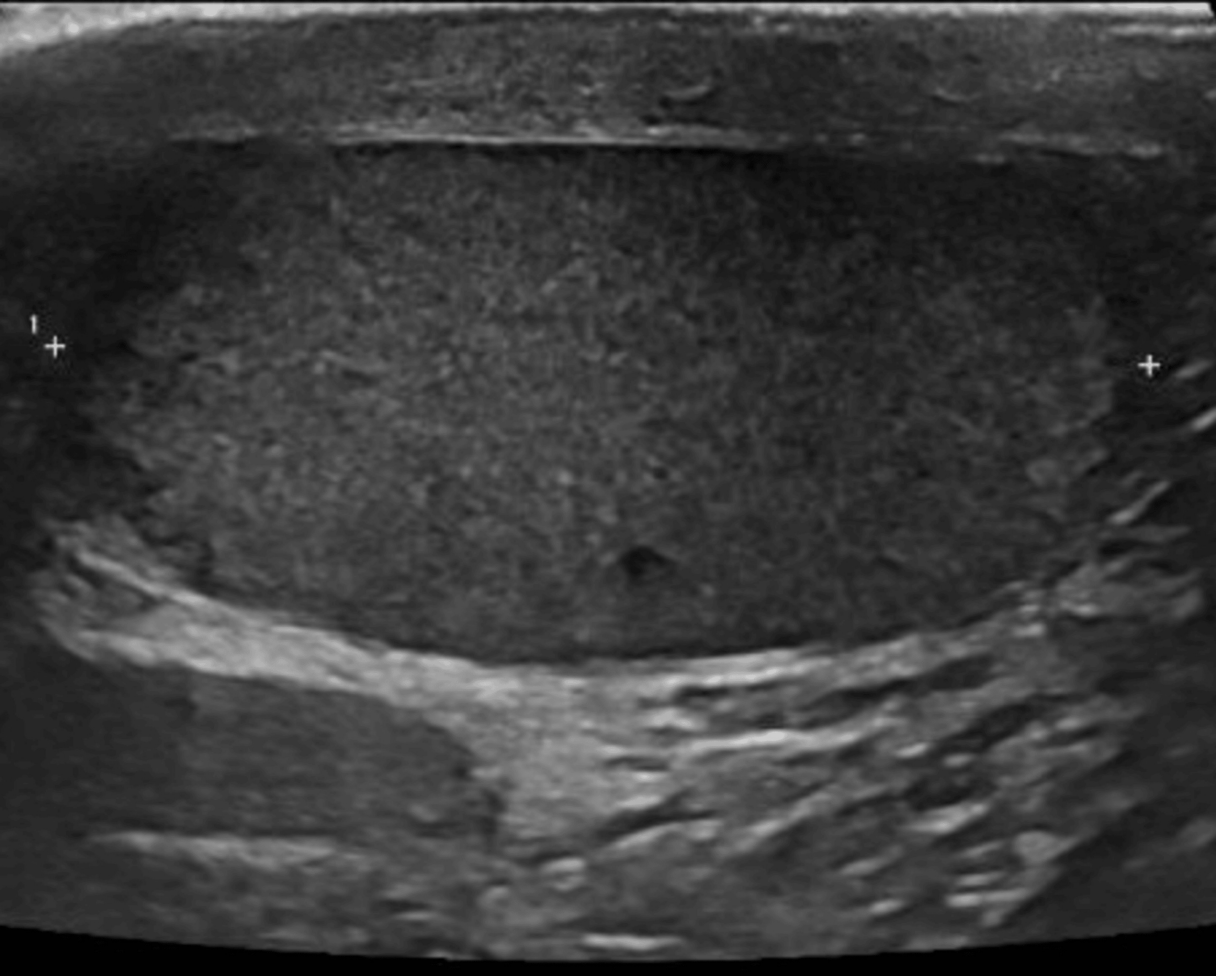
Left (unaffected) testicle with a length of 58 mm.

The left testicle measured 58 mm x 22 mm x 46 mm in size (Figure 2), with normal vasculature and an unremarkable epididymis. Of note, the length of the right testes was 19mm less than that of the unaffected left testicle. On a virtual follow-up following the ultrasound scan, he remained asymptomatic and well.

## Discussion

The authors reviewed the available literature on the PubMed database on July 10, 2024, using the search terms “chickenpox” and “orchitis.” We included all relevant literature describing orchitis secondary to chickenpox and tabulated the cases. Orchitis secondary to chickenpox is a rare occurrence described in only nine previous reports, with our case being the tenth reported case of chickenpox-related orchitis.

The summary of cases from the literature review has been tabulated in Table 1.

**Table 1:** Summary of literature review (nine cases).

Case	Age (years)	Time from rash to orchitis (days)	Affected side	Imaging at point of diagnosis (if done)	Treatment received	Time to clinical resolution (days)	Follow-up
Case 1 [4]	2	5	Left	Not applicable	Pivampicillin for cough Cotrimoxazole for orchitis	15	Not applicable
Case 2 [5]	20	Simultaneous	Bilateral	Not applicable	Not applicable	18	Not applicable
Case 3 [5]	46	9	Right	Not applicable	Penicillin and streptomycin for pneumonia	7	Partially atrophic right testicle (1/3 smaller than the left testicle) On clinical follow-up in 6.5 months
Case 4 [6]	7	5	Left	Not applicable	Procaine penicillin with sulfadiazine Chloramphenicol	5	Complete atrophy on clinical follow-up in six months
Case 5 [7]	2	3	Right	Tc99m scintigraphy - Increased blood flow on dynamic imaging with an area of relative photon deficiency and increased tracer accumulation in the surrounding tissue.	Underwent scrotal exploration -sterile, serous fluid was drained	3	Normal testicle on clinical follow-up in 22 months
Case 6 [7]	5	4	Left	Tc99m scintigraphy - Increased blood flow and tracer accumulation in the left hemiscrotum	Not applicable	3	Normal testicle on clinical follow-up in two months
Case 7 [8]	14	2	Bilateral	Not applicable	Not applicable	7	Normal testicle on clinical follow-up in two months
Case 8 [9]	5	7	Bilateral	Doppler ultrasound - vascular activity in both testicles and epididymis, suggestive of epididymo-orchitis	Intravenous sulbactam-ampicillin, acyclovir during admission and co-amoxiclav on discharge	6	Normal clinical and ultrasound findings on follow-up one week post-discharge
Case 9 [9-10]	20	1	Bilateral	Not applicable	Not applicable	18	Not applicable

**Table 2:** A summary of the nine cases. This table contains  a summary of the cases given in Table 1.

Parameters	Mean value, range and count
Age (in years)	
Mean	13.4 years
Range	2-46 years
Time from rash to orchitis (in days)	
Mean	4 days
Range	0-9 days
Affected side or sides (*n *= 9)	
Right	2
Left	3
Bilateral	4
Time to clinical resolution of orchitis (in days)	
Mean	9.1 days
Range	3-18 days
Follow-up (*n *= 6)	
Partial testicular atrophy	1
Complete testicular atrophy	1
Normal testicle(s)	4

From the information tabulated in Table 1, the following data were extracted and have been summarised in Table 2.

Six of the nine cases were followed up, of which one patient showed partial atrophy, and another showed complete atrophy. Only one patient had an ultrasound scan, albeit one week post-discharge, which might be considered too early.

Infective causes of orchitis include viruses such as mumps, rubella, coxsackievirus, varicella and cytomegalovirus. Bacterial causes include microorganisms commonly implicated in urinary tract infections such as Escherichia coli, Klebsiella pneumoniae and Pseudomonas aeruginosa. However, sexually transmitted diseases such as gonorrhoea (Neisseria gonorrhoeae), chlamydia (Chlamydia trachomatis) and syphilis (Treponema pallidum) are also common in young males. Atypical organisms such as Mycobacterium Avium Complex (MAC), Cryptococcus neoformans, and Candida albicans can also cause orchitis in immunocompromised patients [11].

Chickenpox is a highly infectious disease with an infection rate of up to 90% caused by Varicella Zoster Virus (VZV) - a DNA virus and member of the herpesvirus family. The prevalence of chickenpox is highest between the ages of four to ten years [12] and the transmission of the virus occurs via close personal contact and droplets. An individual with chickenpox remains infectious for 24 hours before the eruption of the rash up until the rash has completely crusted over. The typical progression of the rash seen in chickenpox is from macules to papules and vesicles which develop into pustules and eventually crust in approximately five days [13]. Chickenpox is largely a self-limiting condition, although complications such as pneumonia, septic shock, encephalitis and necrotising fasciitis may require hospital admission [14].

While the relationship between VZV and orchitis have not been studied in depth, our discussion would not be complete without drawing on the current body of knowledge surrounding mumps and orchitis. In contrast to chickenpox, mumps is caused by the paramyxovirus (a single-stranded RNA virus) [15]. Orchitis develops approximately seven days following the onset of parotitis [16] with the management being conservative and the treatment of symptoms as opposed to specific or targeted therapy. Orchitis can occur in up to 30% of post-pubertal men with mumps with up to 30% of all cases of orchitis being bilateral [17]. Evidence suggests that during the acute phase, a reduced testosterone level with elevated luteinizing hormone (LH) and follicle-stimulating hormone (FSH) levels can be found [18]. Testicular atrophy occurred in up to 50% of patients who developed orchitis accompanied by abnormal spermatozoal characteristics and, in turn, subfertility and infertility [16,18]. Of note, impaired fertility in unilateral mumps orchitis is approximately 13% while infertility has been reported in between 30% and 87% of patients with bilateral orchitis secondary to mumps [17]. 

The authors recognised that more data, including the use of antivirals during the acute phase and follow-up data on patients with orchitis secondary to VZV, are needed to determine incidence rates, whether any specific treatment is required and beneficial during the acute phase, and what the long-term sequelae and impact on fertility and testosterone production are, to guide clinicians in managing the condition. The authors also recognised that, similar to orchitis associated with mumps, the limitation and inability to justify a testicular biopsy make understanding the pathophysiology of the condition more challenging.

## Conclusions

Chickenpox-induced orchitis is a rare occurrence. The authors propose that specific treatment with antivirals is not required for chickenpox-induced orchitis and conservative management can be followed with scrotal support and scrotal elevation on rest in conjunction with simple analgesia such as paracetamol and ibuprofen. However, where there is doubt surrounding a superimposed bacterial infection, a course of antibiotics following the department's local antibiotic policy can be adhered to.

The authors recommend that an ultrasound scan of the testes is a suitable modality of following up on such cases as opposed to clinical follow-up alone owing to the potential for partial or total atrophy of the affected testicle. Moreover, confirmation of atrophy allows the patient to be counselled in regards to implications on fertility when the patient enters the reproductive stages of their life as well as testosterone production. 
